# The Annual Meeting of the American Medical Association

**Published:** 1886-06

**Authors:** 


### THE ANNUAL MEETING OF THE AMERICAN MEDICAL ASSOCIATION

Was held in St. Louis on May 4th, 5th, 6th and 7th. The attendance of members was unusually large, and numbered about eleven hundred, including representatives from nearly every state in the Union, and from some of the territories. Rather more than the usual amount of work was done in the general sessions and in the sections. Much good feeling prevailed, contrary to what seems to have been anticipated by some, and but little occurred to mar the pleasure attending the meeting. The provision made by the local committee of arrangements for convenience of registration was exceptionally good, and the entertainments, general and private, tendered the Association were ample to satisfy the most exacting. The following is a list of the officers elected for the year.

OFFICERS FOR 1887:

*President*—Dr. E. H. Gregory, St. Louis, Mo.

*1st Vice-President*—Dr. E. H. Miller, of Stillwater, Minn.

*2nd Vice-President*—Dr. W. B. Welch, of Boonesboro, Ark.

*3d Vice-President*—Dr. William H. Pancoast, of Philadelphia.

*4th Vice-President*—Dr. William C. Wile, of New London, Conn.

*Permanent Secretary*—Dr. William B. Atkinson, of Philadelphia.

*Assistant Secretary*—Dr. J. Nevins Hide, of Chicago, Ill.

*Treasurer*—Dr. R. J. Dunglison, of Philadelphia.

*Librarian*—Dr. C. H. A. Kleinschmidt, of Washington, D. C.

*Committee on Necrology*—Dr. J. M. Toner, of District of Columbia, Chairman; Ala., Jerome Cochran; Ark., C. Watkins; Cal., Beverley Cole; Col., T. H. Hawkins; Conn., Frank H. Whittemore; D. Columbia, C. H. A. Kleinschmidt; Del., Lewis P. Bush; Fla., R. B. Burrows; Ga., R. Battey; Ill., L. H. Montgomery; Ind., J. F. Hibberd; Iowa, J. Williamson; Kan., C. V. Mottram; Ky., R. M. Farleigh; La., J. W. Duprè; Me., A. J. Fuller; Mass., M. G. Parker; Md., T. B. Evans; Mich., S. S. H. French; Miss., B. F. Kittrell; Mo., L. Bremer; Minn., W. W. Mayo; Neb., E. M. Whitten; N. H., J. J. Berry; N. J., I. N. Quimby; N. Y., John Shrady; N. C., Eugene Grissom; Ohio, J. F. Baldwin; Pa., D. G. Brinton; R. I., C. W. Parsons; S. C., R. A. Kinloch; Tenn., J. Y. Crawford;

Tex., J. W. McLaughlin; Ver., E. F. Upham; Va., George B. McCorkle; W. Va., J. H. Pipes; Wis., S. S. Riddell; U. S. Navy, J. C. Speir; U. S. Army, M. K. Taylor; U. S. Mar. Hosp. Service, H. S. Auston; Dak. Ter., J. B. Van Velsor; N. M., G. W. Hansom.

*Committee on State Medicine.*—Ala., G. A. Kitchen; Ark., J. A. Dibrell, Jr.; Cal. F. H. Terrill; Col., P. R. Thomas; Conn., Geo. B. Porter; D. Columbia, J. D. Patterson; Fla., E. T. Sabal; Ga., J. A. McGaston; Ill., J. H. Hollister; Ind., J. H. Beasley; Iowa, P. W. Lewellyn; Kan., S. Schenck; Ky., Wm. Bailey; La., C. W. Day; Mass., M. C. Ledwood, Ira Russell; Md., John Morris; Mich., H. B. Baker; Miss., M. S. Grafft; Mo., J. M. Allen; Minn., W. A. Stenchfield; Neb., A. R. Mitchell; N. H., G. P. Conn; N. J., E. L. B. Godfrey; N. Y., E. S. F. Arnold; N. C., C. J. O. Hagan; Ohio, H. J. Sharp; Pa., W. Sniveley; R. I., W. T. Parker; S. C., C. Kolbrock; Tenn., J. B. Nowling; Tex., C. H. Wilkinson; Vt., H. D. Holton; Va., J. E. Chancellor; W. Va., S. D. Wilson; Wis., C. Alexander; U. S. N., A. L. Gihon; U. S. A., E. McClellan; U. S. M. H. Service, W. H. Long; Dak., J. B. Van Velsor; N. M., W. R. Tipton.

*Members of Judicial Council.*—N. S. Davis, Ill., H. Brown, Ky.; William Brodie, Mich.; D. J. Roberts, Tenn.; R. C. Moore, Neb.; T. A. Foster, Me.; James A. Gray, Ga.

*Trustees of* THE JOURNAL.—P. O. Hooper, Ark.; A. Garcelon, Me.; L. S. McMurtry, Ky.

Place of next meeting, Chicago, Ill., the first Tuesday in June, 1887; Chairman of the Committee of Arrangements, Charles Gilman Smith, M. D., of Chicago.

The next annual meeting will be held in Chicago, in June, 1887.

